# A factor analytic investigation of DSM-5 PTSD symptoms in a culturally diverse sample of refugees resettled in Australia

**DOI:** 10.1186/s13031-018-0155-z

**Published:** 2018-05-23

**Authors:** Philippa Specker, Belinda J. Liddell, Yulisha Byrow, Richard A. Bryant, Angela Nickerson

**Affiliations:** School of Psychology, University of New South Wales, Sydney, NSW 2052 Australia

**Keywords:** Refugees, Asylum-seekers, Post-traumatic stress disorder, Trauma, Confirmatory factor analysis, DSM-5

## Abstract

**Background:**

Refugees and asylum-seekers are often exposed to multiple types of potentially traumatic events (PTEs) and report elevated rates of psychological disorders, including posttraumatic stress disorder (PTSD). Considering this, refugee populations merit continued research in the field of traumatic stress to better understand the psychological impact of these experiences. The symptom structure of PTSD underwent a major revision in the recent formulation in the fifth edition of the *Diagnostic and Statistical Manual of Mental Disorders* (DSM-5), and this reformulation has yet to be comprehensively investigated in the context of PTSD arising from traumatic events experienced by refugees. The current study assessed the construct validity of the DSM-5 PTSD structure in a refugee sample from a variety of cultural backgrounds alongside four alternate models commonly identified in western populations, namely the four-factor Dysphoria model, the five-factor Dysphoric Arousal model, and the six-factor Anhedonia and Externalising Behaviours models.

**Methods:**

A total of 246 refugees settled in Australia were assessed using the Harvard Trauma Questionnaire, to measure exposure to potentially traumatic events (PTEs), and the Posttraumatic Diagnostic Scale, to assess symptoms of PTSD based on DSM-5 criteria. All measures were translated into Arabic, Farsi or Tamil using rigorous translation procedures, or provided in English.

**Results:**

Findings from five confirmatory factor analyses (CFAs) revealed that all models demonstrated acceptable model fit. However, an examination of relative fit revealed that the DSM-5 model provided the poorest fit overall for our sample. Instead, we found preliminary evidence in support of the six-factor Anhedonia model, comprising the symptom clusters of re-experiencing, avoidance, negative affect, anhedonia, dysphoric arousal and anxious arousal, as the superior model for our data.

**Conclusions:**

Our findings offer preliminary support for the applicability of the Anhedonia model to a culturally diverse refugee sample, and contribute to a growing body of studies which indicate that the DSM-5 model may not best represent the symptom structure of PTSD found across non-western conflict-affected populations.

## Background

The psychological presentation of traumatised refugees and asylum seekers is complex and presents a global challenge to public health [1]. There are currently an estimated 65.6 million refugees, asylum seekers and internally displaced people worldwide and this number is growing [2]. Refugees and asylum-seekers are often exposed to multiple types of potentially traumatic events (PTEs) and report elevated rates of psychological disorders, including posttraumatic stress disorder (PTSD) [3–5]. Despite this, refugees remain under-represented in research on traumatic stress. Since the introduction of PTSD in the third edition of the *Diagnostic and Statistical Manual of Mental Disorders* (DSM-III) [6], the symptom structure of PTSD has been investigated using samples predominantly from high-income western countries, such as single incident trauma survivors and military personnel [7]. The symptom structure of PTSD underwent a major revision in the recent formulation in DSM-5 [8], and this reformulation has yet to be comprehensively investigated in the context of PTSD arising from refugee experiences. Theorists and researchers have questioned the capacity of DSM-derived PTSD models to capture the psychological sequelae arising from experiences of persecution and/or displacement in non-western populations [9–11]. To investigate this, the current study assessed the construct validity of the DSM-5 PTSD structure, alongside alternate models commonly identified in western populations, in a refugee sample from non-western backgrounds.

### The DSM-5 and competing PTSD models

The underlying factor structure of PTSD symptoms outlined in the DSM-IV [12] has been the topic of longstanding academic debate [13]. The DSM-IV model of PTSD, which largely resembles the DSM-III model, comprised 17 symptoms across three factors: re-experiencing, avoidance/numbing, and arousal [12]. In order to meet diagnostic criteria for PTSD, DSM-IV required the individual to have experienced a traumatic event, and to endorse at least one re-experiencing symptom, three avoidance/numbing symptoms, and two arousal symptoms. Prior to the substantial reformulation of PTSD for the DSM-5, numerous confirmatory factor analytic studies (CFA) consistently identified three alternate PTSD models as superior to the DSM-IV tripartite model: the four-factor Emotional Numbing model [14], the four-factor Dysphoria model [15], and the five-factor Dysphoric Arousal model [16]. The Emotional Numbing model conceptualises avoidance and numbing symptoms as two separate factors (see Table 1), drawing on evidence that these symptoms tend not to cluster together [17, 18]. The Dysphoria model combines numbing symptoms and three symptoms from the arousal cluster (sleeping difficulties; irritability and anger; concentration difficulties) into a single factor called dysphoria, which represents symptoms of general distress that are not necessarily specific to PTSD (see Table 1). This reformulation drew on evidence demonstrating that the hypervigilance and exaggerated startle response symptoms were distinct from the three remaining symptoms of the arousal factor [19]. Finally, Elhai et al. [16] developed the Dysphoric Arousal model, which combined elements of the Emotional Numbing and Dysphoria models. This model separates avoidance and numbing symptoms into two distinct factors, and also separates the arousal symptom cluster into anxious arousal and dysphoric arousal symptoms (see Table 1). Despite widespread support for all three competing models as better representations of PTSD than the DSM-IV model, empirical evidence has generally shown that the five-factor Dysphoric Arousal model is superior overall [16, 20].

**Table 1:** Symptom mapping of five competing PTSD models

PTSD Symptoms (DSM-5)	DSM-5 (4-factor)	Dysphoria (4-factor)	Dysphoric Arousal (5-factor)	Anhedonia (6-factor)	Externalising Behaviours (6-factor)
B1: Recurrent thoughts of trauma	R	R	R	R	R
B2: Recurrent dreams of trauma	R	R	R	R	R
B3: Flashbacks	R	R	R	R	R
B4: Psychological cue reactivity	R	R	R	R	R
B5: Physiological cue reactivity	R	R	R	R	R
C1: Avoidance of thoughts of trauma	AV	AV	AV	AV	AV
C2: Avoidance of reminders of trauma	AV	AV	AV	AV	AV
D1: Trauma-related amnesia	NAMC	D	NAMC	NA	N
D2: Negative beliefs^a^	NAMC	D	NAMC	NA	N
D3: Distorted blame^a^	NAMC	D	NAMC	NA	N
D4: Persistent negative emotional state^a^	NAMC	D	NAMC	NA	N
D5: Diminished interest in activities	NAMC	D	NAMC	An	N
D6: Feelings of detachment from others	NAMC	D	NAMC	An	N
D7: Inability to experience positive emotions	NAMC	D	NAMC	An	N
E1: Irritability or anger	A	D	DA	DA	EB
E2: Reckless or self-destructive behaviour^a^	A	D	DA	DA	EB
E3: Hypervigilance	A	A	AA	AA	AA
E4: Exaggerated startle response	A	A	AA	AA	AA
E5: Difficulty concentrating	A	D	DA	DA	DA
E6: Sleeping difficulties	A	D	DA	DA	DA

*A* = alternations in arousal and reactivity, *AA* = anxious arousal, *An* = anhedonia, *AV* = avoidance, *D* = dysphoria, *DA* = dysphoric arousal, *EB* = externalising behaviours, *H* = Hyperarousal, *N* = numbing, *NA* = negative affect, *NAMC* = negative alterations to mood and cognitions, *R* = re-experiencing. ^a^ = symptoms in DSM-5 criteria that were not present in the DSM-IV criteria

The DSM-5 [8] fundamentally revised the symptomology of PTSD from the DSM-IV model to comprise 20 symptoms across four factors of PTSD: re-experiencing, avoidance, negative alterations to mood and cognitions (NAMC), and alterations in arousal and reactivity. In the DSM-5, a PTSD diagnosis requires the individual to endorse at least one re-experiencing symptom, one avoidance symptom, two NAMC symptoms, and two alterations in arousal and reactivity symptoms. The DSM-5 model thus separates the avoidance and numbing symptoms into two distinct clusters, in line with the Emotional Numbing model, to create a new cluster, NAMC, that comprises numbing symptoms as well as three new symptoms (D2-D4: negative beliefs; distorted blame; persistent negative emotional state). Also, the DSM-5 adds a new symptom of reckless or self-destructive behaviour (E2) to the arousal and reactivity symptom cluster (see Table 1). These major changes have prompted the re-investigation of the latent structure of PTSD and the emergence of additional alternate models. These new models include the Anhedonia model [21] and the Externalising Behaviours model [22] alongside DSM-5 versions of the Dysphoria and Dysphoric Arousal models. The six-factor Anhedonia model separates the arousal symptom cluster into anxious and dysphoric arousal, in line with the DSM-IV derived Dysphoric Arousal model. In addition, this model separates the NAMC symptom cluster into negative affect and anhedonia, where anhedonia is considered to be a deficit in one’s capacity to experience positive affect (see Table 1). This novel reformulation of the NAMC factor was motivated by research indicating the conceptual and empirical distinctness of positive affect from negative affect [23–25]. The six-factor Externalising Behaviours model, akin to the Anhedonia and Dysphoric Arousal models, separates the arousal symptom cluster into anxious and dysphoric arousal but also moves two symptoms, irritability or anger (E1) and reckless or self-destructive behaviour (E2), out of the dysphoric arousal cluster and into a new factor called externalising behaviours (see Table 1). This new cluster was created to represent self-initiating aggressive behaviours that potentially signify emotion dysregulation, and was based on Tsai et al.’s [22] theorising that such behaviours are distinct to the remaining dysphoric arousal symptoms (difficulty concentrating, E5; sleeping disturbances, E6).

To date, CFA studies have found that the DSM-5 model provides adequate to good fit in trauma-exposed samples [7]. However, Armour et al.’s [7] systematic review of the CFA literature on DSM-5 PTSD symptoms outlined an emerging trend in the more recent CFA studies whereby models that specify more factors tend to demonstrate better fit. In particular, preliminary empirical investigations of the newly proposed six-factor models have found them to be superior to four- and five-factor models, including the DSM-5 model [21, 22]. Further, the only two studies, to our knowledge, that have directly compared the two six-factor models both found the Anhedonia model to be superior [26, 27].

### Factor structure of PTSD among conflict-affected populations

Validation of the symptom structure of PTSD across non-western populations is a necessary prerequisite to establishing a culturally robust model for understanding traumatic stress [28]. Yet, to date, research on PTSD, has overwhelmingly relied on western samples [7]. While this represents a useful starting point for understanding traumatic stress, it is important that further research captures the diversity of traumatic experiences that occur globally. In particular, refugee populations are exposed to a wide variety of traumatic events that are distinct from western experiences of trauma. For instance, refugees often report multiple, prolonged and severe traumatisation, including torture, political persecution, and traumatic bereavement [29, 30]. Another distinguishing feature of resettled refugee populations is their experience of post-migratory stress, which has been shown to strongly influence symptoms of traumatic stress [31]. Accordingly, experiences of persecution and displacement are characteristic of refugee populations and stand in contrast to traumatic experiences commonly studied in western populations. As such, understanding the core ways that experiences of persecution and displacement influence the symptom structure of PTSD is uniquely valuable in advancing our conceptualisation of PTSD in refugees and asylum-seekers and assisting a population in great need of treatment interventions.

While there is a small body of literature investigating the factor structure of the DSM-IV model with non-western refugee and post-conflict samples [11, 32–35], only two studies to date that have examined the DSM-5 model in refugee samples [9, 36]. Schnyder et al. [36] investigated the latent structure of DSM-5 PTSD in a sample of 134 refugees undergoing treatment for PTSD in Switzerland. While the DSM-5 model demonstrated good fit, no alternate model based on DSM-5 criteria was tested so it cannot be determined whether the DSM-5 model was the most appropriate model for Schnyder et al.’s sample. Additionally, Michalopoulos et al. [9] investigated PTSD model fit in three non-western samples from low or middle income countries: 974 torture survivors in Iraq, 1189 sexual violence survivors in the Democratic Republic of Congo (DRC), and 535 Burmese refugees in Thailand. Although Michalopoulos et al. assessed PTSD symptoms using a measure that was based on DSM-IV criteria, they did examine an approximated DSM-5 model. Of interest, while the DSM-5 model demonstrated adequate to good fit among the DRC and Burmese samples, it provided the poorest relative fit compared to the DSM-IV, Dysphoria, and Emotional Numbing models. Instead, the Emotional Numbing model was superior for the DRC sample and the Dysphoria model best represented the data from the Burmese sample. Further, the DSM-5 model, along with all alternate models, did not adequately represent PTSD symptoms in the Iraqi sample. Importantly, this small body of empirical research highlights inconsistencies in the validity of the DSM-5 PTSD model when applied to non-western samples, and offers preliminary insight into potential limitations of the DSM-5 model in accurately representing the symptom structure of PTSD among refugee and post-conflict populations. As such, further research using refugee samples is required to clarify the validity of western-derived PTSD models, particularly the DSM-5 model, for individuals exposed to persecution and displacement.

### The current study

The current study employed a culturally diverse refugee sample to examine the construct validity of the DSM-5 PTSD model, alongside four competing models: the four-factor Dysphoria model, the five-factor Dysphoric Arousal model, and the six-factor Anhedonia and Externalising Behaviours models. To our knowledge, this is the first study to investigate the validity of the Dysphoria and Dysphoric Arousal models with a refugee sample, based on DSM-5 PTSD criteria, as well as the first to assess the appropriateness of the newly proposed Anhedonia and Externalising Behaviours models in representing the structure of PTSD symptoms in refugees. Based on previous findings from studies with non-refugee samples, we hypothesise that the Anhedonia model will evidence the best fit with the current sample.

## Methods

### Participants

A total of 246 refugees and asylum-seekers settled in Australia participated in the current study. Participants were recruited via advertisements at a number of refugee services and organizations as well as through social media, community groups, newsletters and radio stations. Potential participants who had registered their interest in the study then completed a brief online survey or were contacted by telephone to assess eligibility. Eligibility criteria required participants to: a) be over the age of 18 years, b) have a refugee or asylum-seeker background, c) be literate in either Arabic, Farsi/Persian, Tamil or English, d) be residing in the Australian community, and e) have arrived in Australia after 1st January 2011. In total, 163 (66.3%) participants completed the questionnaire online and 83 (33.7%) participants completed pen and paper versions of the study. Sociodemographic characteristics of the sample are presented in Table 2. One hundred and eleven (45.5%) participants had secure visa status, i.e. held permanent visas, and 132 (55.5%) had insecure visa status, i.e. held temporary, bridging or expired visas, or had no visa. Seventy-eight (31.7%) participants completed the survey in Arabic, 70 (28.5%) in Farsi/Persian, 66 (26.8%) in Tamil, and 32 (13%) in English.

**Table 2:** Sociodemographic characteristics of refugees settled in Australia

Variable	*M* or *n*	*SD* or *%*
Male gender	111	45.1%
Age, years	38.25	11.86
Country of Birth		
Iraq	75	30.5
Iran	71	28.9
Sri Lanka	68	27.6
Afghanistan	8	3.3
Others	24	9.7
Ethnicity		
Tamil	69	28
Iranian	47	19.1
Iraqi	29	11.8
Chaldean	23	9.3
Assyrian	14	5.7
Arab	12	4.9
Persian/Farsi	10	4.1
Hazara	8	3.3
Others	34	13.8
Marital Status		
Married	145	58.9
Single	65	26.4
Separated/Divorced	16	6.5
In a Relationship/Cohabiting/Defacto	13	5.2
Widowed	6	2.4
Educational Attainment		
Little or no formal education	10	4.1
Completed primary	28	11.4
Completed high school	99	40.2
Completed university	76	30.9
Completed vocational training	28	11.4
Months in Australia	29.02	12.34
Visa Status		
Secure	111	45.5
Insecure	135	55.5

*N* = 246

### Measures

Accredited interpreters translated all measures used in the current study into Arabic, Farsi and Tamil. Following this, blinded back translation procedures were employed [37]. The research team, in conjunction with the interpreters, rectified any discrepancies that emerged from this process.

#### The Harvard trauma questionnaire (HTQ) [38]

The HTQ is a 16-item self-report measure used to index exposure to different types of potentially traumatic events (PTEs), including lack of food or water, forced isolation, serious injury and torture. For each PTE, participants were asked to indicate whether they had: *experienced myself, witnessed this happening to others, learned about this happening to a friend/family member,* or *none of the above*, where more than one response could be selected. For the purposes of the present study, responses were then dichotomised, such that an item was considered as endorsed if participants indicated they had *experienced* and/or *witnessed* the PTE. Dichotomised responses were subsequently summed to produce a total count of exposure to PTEs.

#### Posttraumatic diagnostic scale (PDS) [39]

The PDS is a 20-item self-report measure used to assess DSM-IV symptoms of PTSD. We adapted items in the original scale according to DSM-5 PTSD criteria. Each scale item corresponds to a DSM-5 PTSD symptom, such as: having intrusive thoughts about the traumatic event, feeling emotionally numb, and being overly alert. Additional items assessed persistent negative beliefs, persistent extreme blame of the self or others, strong negative emotional state, and risk-taking behaviour. Participants were asked to rate, on a 4-point scale, how frequently they had experienced each symptom over the preceding month. Responses ranged from 0 “*not at all or only once”* to 3 “*5 or more times a week/ almost always”*. A DSM-5-derived algorithm was used to determine probable PTSD diagnosis in this study, which required experiencing at least one traumatic event and rating at least one intrusion symptom, one avoidance symptom, two NAMC symptoms, and two alterations to arousal and reactivity symptoms as 2 or greater.

### Procedure

Eligible participants were emailed the link to an online version of the questionnaire via Key Survey Version 8.6 or posted a pen and paper version of the questionnaire. Participants first provided demographic details before completing a battery of measures, which included the HTQ and PDS. Upon completion, participants received a $25 (US$20) gift voucher. Ethics approval for the current study was provided by the University of New South Wales Research Ethics Committee.

### Data analysis

Using Preacher and Coffman’s [40] software to calculate statistical power for RMSEA, it was found that a minimum sample of 131 was required to achieve 80% power when degrees of freedom are 155 and RMSEA is 0.05. As such, our sample size provided sufficient statistical power to conduct the CFAs. Five CFAs were conducted to assess the fit of the data to the 4-, 5- and 6-factor models. Also, a CFA of a one-factor model, where all symptoms were specified as a single factor, was conducted and reported. All analyses were conducted using Mplus version 8 [41]. In line with Flora and Curran’s [42] and Wirth and Edwards’ [43] recommendations for estimating ordinal data, mean and variance-adjusted weighted least squares (WLSMV) estimation was used to generate model fit indices [44]. The missing data rates for the items used in the CFAs was less than 5% (ranging from 0 to 3.3%), and the default option, pairwise present analysis, was employed for missing data [41]. Also, two participants were not included in analyses as their data was missing across all variables. The root mean squared error of approximation (RMSEA) [45], comparative fit index (CFI) [46] and Tucker-Lewis Index (TLI) [47] were used to evaluate goodness of fit. Although reported, the χ^2^ statistic was not used to evaluate model fit due to its over-sensitivity to sample size [48]. However, mean and variance adjusted χ^2^ difference tests were used to statistically compare nested models to determine relative model fit. Each model was evaluated according to the benchmarks proposed by Yu [49], whose Monte Carlo simulation studies with categorical data suggest that an RMSEA below 0.05, CFI greater than 0.96 and TLI greater than 0.95 indicate good model fit for samples of approximately 250.

## Results

### Exposure to PTEs and probable PTSD diagnosis

Responses on the HTQ revealed that participants in this sample had been exposed to multiple types of PTEs. On average, participants had experienced 5.68 (*SD* = 4.82, range: 0–16) types of PTEs, with the vast majority (*n* = 180, 81.1%) reporting exposure to at least one type of PTE. The frequency of exposure to PTEs are summarised in Table 3. Participants most commonly experienced lack of food or water (*n* = 139, 56.5%) and being close to death (*n* = 122, 50%). Additionally, more than one third of the sample had experienced imprisonment (*n* = 98, 40.2%) and/or torture (*n* = 88, 36.1%), and just under one fifth were survivors of rape or sexual abuse (*n* = 45, 18.7%). A total of 51 (20.8%) participants were identified as having a probable diagnosis of PTSD.

**Table 3:** Frequency of Exposure to Potentially Traumatic Events

Trauma	*N*	*%*
Lack of food or water	139	56.5
Being close to death	122	50
Ill health without access to medical care	114	46.5
Lack of shelter	113	46.1
Serious injury	104	43.9
Forced separation from family members	103	42.4
Imprisonment	98	40.2
Combat situation	97	39.9
Torture	88	36.1
Forced isolation from others	86	35.5
Lost or kidnapped	71	29.2
Unnatural death of family or friend	69	28.4
Brain washing	63	26.5
Murder of family or friend	54	22.3
Murder of stranger or strangers	46	19.2
Rape or sexual abuse	45	18.7

*N* = 246

### Confirmatory factor analyses

#### Goodness of fit and model comparisons

Goodness of fit indices for each of the competing models are presented in Table 4. All models, except the one-factor model, demonstrated good fit across CFI and TLI. However, RMSEA across all models was higher than the proposed cut off of 0.05, with the one-factor model being notably poor (0.104) and all remaining models providing similar, yet less than adequate, RMSEAs ranging from 0.073–0.075.

**Table 4:** Model goodness of fit indices

	WLSMV- χ^2^	*df*	CFI	TLI	RMSEA (90% CI)
1 DSM 5 model	387.086*	164	.979	.976	.075 (.065–.084)
2 Dysphoria model	382.629*	164	.980	.976	.074 (.064–.084)
3 Dysphoric Arousal model	367.390*	160	.981	.977	.073 (.063–.083)
4 Anhedonia model	355.838*	155	.981	.977	.073 (.063–.083)
5 Externalising Behaviours model	366.487*	155	.980	.976	.075 (.065–.085)
6 One-factor model	617.857*	170	.958	.954	.104 (.095–.113)

*df* = degrees of freedom; *CFI* = comparative fit index; *TLI* = Tucker-Lewis index; *RMSEA* = root mean squared error of approximation; *CI* = confidence interval

*Significant relative to degrees of freedom, *p* < .001

To determine relative model fit, χ^2^ difference tests between nested models were conducted, and the results are summarised in Table 5. Findings showed that all four-, five-, and six-factor models were significantly better than the one-factor model. Further, the five-factor Dysphoric Arousal model and the six-factor models were significantly better than both the four-factor DSM-5 and Dysphoria models. Of interest, only the six-factor Anhedonia model, and not the six-factor Externalising Behaviours model, was significantly better than the five-factor Dysphoric Arousal model. Overall, these findings provide preliminary evidence that the six-factor Anhedonia model demonstrated superior fit.

**Table 5:** χ^2^ difference test comparing nested models

Models	Δχ^2^ (*df*)	*p*-value
Model 1 vs. Model 3	22.41 (4)	<.001
Model 1 vs. Model 4	38.74 (9)	<.001
Model 1 vs. Model 5	30.67 (9)	<.001
Model 2 vs. Model 3	19.57 (4)	<.001
Model 2 vs. Model 4	35.37 (9)	<.001
Model 2 vs. Model 5	27.78 (9)	.001
Model 3 vs. Model 4	15.77 (5)	<.010
Model 3 vs. Model 5	6.78 (5)	.238
Model 6 vs. Model 1	123.33 (6)	<.001
Model 6 vs. Model 2	124.28 (6)	<.001
Model 6 vs. Model 3	157.94 (10)	<.001
Model 6 vs. Model 4	190.00 (15)	<.001
Model 6 vs. Model 5	186.35 (15)	<.001

*N* = 244

#### Factor loadings and Intercorrelations

Following tentative preliminary evidence in support of the applicability of the Anhedonia model to our data, factor loadings and correlations were inspected for this model. Standardised factor loadings for the Anhedonia model are presented in Table 6. All items had high factor loadings, greater than .70. Of interest, DSM-5 symptoms B4 (psychological reactivity to traumatic reminders) and B5 (physiological reactivity to traumatic reminders) were particularly characteristic of the re-experiencing factor with factor loadings of .97 and .92 respectively. Also, factor loadings across the avoidance factor were very high, with symptoms C1 (avoidance of traumatic thoughts) and C2 (avoidance of traumatic reminders) both loading at .90. Similarly, both symptoms for the anxious arousal factor, E3 (hypervigilance) and E4 (exaggerated startle response), had very high factor loadings of .88 and .92 respectively.

**Table 6:** Standardised factor loadings for the Anhedonia model

PTSD Symptoms	Anhedonia model
Re-experiencing	
B1: Recurrent thoughts of trauma	.84
B2: Recurrent dreams of trauma	.87
B3: Flashbacks	.87
B4: Psychological cue reactivity	.97
B5: Physiological cue reactivity	.92
Avoidance	
C1: Avoidance of thoughts of trauma	.90
C2: Avoidance of reminders of trauma	.90
Negative Affect	
D1: Trauma-related amnesia	.70
D2: Negative beliefs	.78
D3: Distorted blame	.77
D4: Persistent negative emotional state	.87
Anhedonia	
D5: Diminished interest in activities	.78
D6: Feelings of detachment from others	.80
D7: Inability to experience positive emotions	.85
Dysphoric Arousal	
E1: Irritability or anger	.83
E2: Reckless or self-destructive behaviour	.81
E5: Difficulty concentrating	.85
E6: Sleeping difficulties	.88
Anxious Arousal	
E3: Hypervigilance	.88
E4: Exaggerated startle response	.92

*N* = 244. All factor loadings are statistically significant (*p* < .001)

Factor intercorrelations for the Anhedonia model are summarised in Table 7. Inspection of factor intercorrelations for the Anhedonia model revealed that Negative Affect had a correlation of 0.957 with Anhedonia and a correlation of 0.956 with Dysphoric Arousal. Following the recommendations of Brown [44] and Kline [48], this pattern of high correlations prompted consideration of an alternate model where Anhedonia and Dysphoric Arousal were collapsed onto Negative Affect to create a single factor. However, this four-factor model was identical to the factor structure of the Dysphoria model and a previously conducted χ^2^ difference test found that the Anhedonia model demonstrated significantly better fit than the nested Dysphoria model. As such, the Anhedonia model, despite some high factor intercorrelations, still appears to fit the data better than the Dysphoria model.

**Table 7:** Factor intercorrelations for the six-factor Anhedonia model

	R	AV	NA	An	DA	AA
R	1					
AV	.817	1				
NA	.858	.889	1			
An	.863	.882	.957	1		
DA	.801	.813	.956	.899	1	
AA	.786	.770	.918	.801	.863	1

*N* = 244. *AA* = anxious arousal, *An* = anhedonia, *AV* = avoidance, *DA* = dysphoric arousal, *NA* = negative affect, *R* = re-experiencing. All correlations are statistically significant (*p* < .001)

## Discussion

The current study examined the construct validity of the DSM-5 PTSD model, alongside four competing models identified in the literature, in capturing the trauma-related psychopathology of those exposed to persecution and displacement. To our knowledge, this is the first study to investigate with a refugee sample the validity of the Dysphoria and Dysphoric Arousal models, based on DSM-5 criteria, in addition to the newly proposed Anhedonia and Externalising Behaviours models. Findings from five CFAs revealed that all models of interest, including the DSM-5 model, demonstrated good fit across CFI and TLI but higher than desirable RMSEA. However, relative to the alternate four, five and six-factor models examined, the DSM-5 model was the poorest fitting model for our data. Moreover, our study found preliminary evidence in support of the Anhedonia model as the best fitting model for our sample of traumatised refugees.

Our findings add to a small, but growing, body of research on the symptom structure of PTSD within culturally diverse refugee and post-conflict samples. With regards to the DSM-5 model, our findings accord with previous research [9, 36]. Schnyder et al. [36] found that the DSM-5 model produced adequate to good model fit among treatment-seeking refugees in Switzerland. This is consistent with our study where the DSM-5 model demonstrated adequate fit for our refugee sample. Importantly, however, the present study found that the DSM-5 model had the poorest relative fit for our sample compared to all four of the competing models that we tested: the four-factor Dysphoria model, five-factor Dysphoric Arousal model, and six factor Anhedonia and Externalising Behaviours models. This additional finding is not necessarily inconsistent with Schnyder et al.’s findings as alternate PTSD models based on DSM-5 criteria were not tested, so it is entirely possible that their sample would have yielded similar patterns of relative fit if alternate DSM-5 models had been tested. Notably, our findings are broadly consistent with Michalopoulos et al.’s [9] study, which assessed relative model fit of the DSM-IV model, DSM-IV Dysphoria model, and DSM-IV Emotional Numbing model against an approximation of the DSM-5 model among three culturally diverse samples from low or middle income countries: sexual assault survivors from the DRC, Burmese refugees in Thailand, and Iraqi torture survivors. Michalopoulos et al. found that although their approximation of the DSM-5 model evidenced adequate to good fit for their DRC and Burmese samples, it demonstrated poor fit for Iraqi survivors of torture. Moreover, the DSM-5 model was not the best fitting model for any of the samples. Instead, the best fitting model varied across samples: the Emotional Numbing model demonstrated superior fit for the DRC sample, the Dysphoria model was most appropriate for the Burmese sample, and none of the models tested adequately represented the Iraqi sample. The authors acknowledged that time since trauma exposure may have been a factor explaining the poor fit of the models to the Iraqi sample, as the height of trauma exposure for the Iraqi participants was approximately 20 years prior to data collection. However, Michalopoulos et al. did not assess more complex PSTD models, such as the newly proposed Anhedonia model, which may have been more appropriate for this sample. Taken together, our findings build on extant research to demonstrate that the DSM-5 model, although generally producing adequate fit, was not the best representation of the latent structure of PTSD when applied to this culturally-diverse refugee sample.

In the current study, the six-factor Anhedonia model fit the data better than the four-factor DSM-5 and Dysphoria models, the five-factor Dysphoric Arousal model, and the six-factor Externalising Behaviours model. This finding replicates previous research with non-refugee samples that found the Anhedonia model to be superior to all other four-, five- and six-factor models [26, 27] and conforms to a trend in previous research where best fitting models tend to specify more factors [7]. However, it should be noted that two factors of the Anhedonia model, the Avoidance and Anxious Arousal factors, comprise only two items. This may be problematic from a statistical standpoint as models that specify factors with less than three indicators may result in an under-identification of the model and inaccurate or unstable parametric estimates in CFA [7, 48]. While this is a notable statistical limitation of the Anhedonia model, it is important to consider the ubiquity of this problem as many of the DSM-5-derived models for PTSD include at least one factor (Avoidance) that is only specified by two items, based on the DSM-5 symptom criteria in which only two symptoms are specified in this cluster. Moreover, other frameworks, such as the *International Statistical Classification of Diseases and Related Health Problems* (ICD) are moving towards more parsimonious PTSD models that include fewer factors with fewer items in each factor. For example, the proposed PTSD diagnosis for the ICD-11 comprises three factors with two symptoms each [50]. As such, there is a need to reconcile this statistical limitation with the value of theoretically-derived parsimonious models that consider the availability of clinical resources in the field. A possible solution for future investigations of such models, offered by Marsh and colleagues [51], could be to use larger samples, of 400 participants or greater, in analyses to ensure fully valid solutions.

The Anhedonia model, comprising intrusion, avoidance, negative affect, anhedonia, dysphoric arousal and anxious arousal, deviates from the DSM-5 model in two key ways. First, it divides symptoms of arousal into dysphoric arousal, comprising symptoms of irritability or anger (E1), reckless or self-destructive behaviour (E2), difficulty concentrating (E5), and sleeping difficulties (E6), and anxious arousal, comprising symptoms of hypervigilance (E3) and exaggerated startle response (E4) [21]. This separation is supported by CFA studies that demonstrated that anxious and dysphoric arousal were distinct constructs among representative samples from Australia and the United States [52], Malaysian tsunami survivors [53], terrorist attack first-responders [54], and adolescent earthquake survivors [55]. Notably, while Liu et al. [21] found that the two factors of dysphoric and anxious arousal were strongly correlated (.97), this correlation is lower in our sample (.86), suggesting that the two symptom clusters, although related, are distinct from one another. Second, the Anhedonia model divides NAMC symptoms into negative affect and anhedonia (deficit in experiencing positive affect), which is supported by theoretical and empirical evidence that alterations to positive and negative affect represent distinct constructs in mood and anxiety disorders [24, 25, 56]. It is notable that the fit of the Anhedonia model, which was the only model to differentiate between symptoms of negative affect and anhedonia, was superior to all the other models that were examined. While negative affect yielded high factor correlations with anhedonia and dysphoric arousal in our sample, this is consistent with previous research [21, 57]. Moreover, the collapsing of these symptoms into a single factor produced the Dysphoria model, which demonstrated a significantly worse fit compared to the Anhedonia model. As such, negative affect and anhedonia appear to represent two distinct constructs of DSM-5-defined PTSD for our sample. Further, while the Anhedonia model evidenced some high factor intercorrelations, a single factor model was tested and demonstrated unacceptably poor fit statistics and significantly poorer fit relative to the Anhedonia model, which suggests that a single factor model does not best fit the underlying factor structure of PTSD in this sample.

In the current study, all PTSD symptoms displayed relatively high factor loadings on their corresponding factors in the Anhedonia model. In particular, we found that psychological and physiological reactivity to traumatic reminders (B4 and B5) displayed very high factor loadings on the re-experiencing factor (.97 and .92 respectively). Similarly, Schnyder et al.’s [36] study also evidenced high factor loadings across these symptoms (.92 and .89 respectively) in a sample of treatment-seeking refugees. This finding may be reflective of the unique experiences of refugees who are exposed to a complex constellation of cumulative interpersonal trauma, uncertainty and on-going stressors. First, our sample was exposed to a very high number of interpersonal traumatic events, i.e. trauma that is perpetrated by another human, and previous research has found distress to reminders (as well as intrusive memories) are significantly elevated among survivors of interpersonal trauma compared to non-interpersonal trauma [58]. In addition, many refugees regularly encounter highly salient reminders of traumatic events by virtue of exposure to information about ongoing persecution and conflict in the home country (i.e., via media reports and contact with family in the home country), which is likely to contribute substantially to psychological and physiological distress. As such, reactivity to traumatic reminders may be especially characteristic of symptoms of re-experiencing for refugees. Following this, refugees may attempt to avoid thinking about or talking about past traumatic experiences, which may account for the high factor loadings of avoidance of traumatic thoughts and reminders (C1 and C2) in this study. Further research is required to elucidate the phenomenology of these symptoms and their inter-relationships in trauma-affected refugees.

Our sample also displayed very high factor loadings for the symptoms of hypervigilance and an exaggerated startle response (E3 and E4) on the anxious arousal factor, which may have been due to the high rates of prolonged and repeated exposure to interpersonal traumatic events such as torture, combat, kidnapping and sexual violence. This would be consistent with longitudinal research by Forbes et al. [58] that found significantly higher rates of both hypervigilance and an exaggerated startle response symptoms among survivors of interpersonal trauma compared to those exposed to non-interpersonal trauma.

Previous CFA studies using non-refugee samples repeatedly found relatively low factor loadings for symptoms of reckless or self-destructive behaviours [21, 57, 59], yet, this was not the case for our sample where factor loadings were high (.81). Notably, this symptom also produced a high factor loading for Schnyder et al.’s [36] refugee sample (.74). This suggests that symptoms of reckless or self destructive behaviours may be more relevant to the presentation of PTSD within refugee samples. Indeed, refugees, who experience multiple and prolonged traumatisation, often present with complex reactions to traumatic events, which can manifest as reckless behaviour [30]. This finding calls for further investigation to identify what kinds of reckless behaviours trauma-affected refugees may be especially likely to engage in. Studies that have investigated this further have differed substantially in how they define these behaviours. For example, Michalopoulos et al. [9] operationalised reckless behaviour as “drinking too much alcohol” in their Iraqi and Burmese samples and although the authors did not report factor loadings, they found that this was the least frequently endorsed item for both samples. An alternative conceptualization of reckless behaviours that may be especially relevant is self-harming behaviours, which have been found to be elevated among refugee populations [60, 61]. Further research is required to elucidate the specific manifestations of this symptom amongst trauma-affected and displaced refugee populations.

Symptoms relating to anger (E1), negative beliefs (D2), distorted blame (D3), and persistent negative emotional state (D4) also displayed relatively high factor loadings in our sample, ranging from .77 to .87. This finding also aligns with previous research that found that refugees displayed different forms of emotion dysregulation in reaction to traumatic events, such as excessive guilt, self-blame and outbursts of anger [30]. In light of this, the decision to broaden the scope of PTSD in the DSM-5 to include the new symptoms of reckless or destructive behaviours (E2), negative beliefs (D2), distorted blame (D3), and persistent negative emotional state (D4), may be particularly pertinent to the clinical presentation of PTSD amongst refugees.

Several limitations of the current study should be acknowledged. First, our sample comprised participants from a number of different cultural backgrounds. Although this is an ecologically valid representation of the cultural diversity inherent in global refugee populations, it is possible that important cultural differences specific to a single group may have been masked in the current study. Moreover, the cultural and linguistic variation present in our sample may have influenced the model fit for our data. However, notwithstanding this notable limitation, the aim of the present study was to investigate the phenomenology of PTSD among a culturally diverse sample of people who have experienced persecution and displacement, in order to reflect the global refugee population. As such, we chose to analyse our refugee sample collectively, rather than according to specific sub-populations, as a way to adequately represent, and understand, the universal features of traumatic stress. A second limitation was that self-report questionnaires were used to assess PTSD symptoms. Although self-rated PTSD symptom scores are strongly correlated to clinician-rated PTSD symptom scores [62], clinician-administered structured interviews provide additional standardised information regarding symptom severity and clinical impairment, which was not measured in the current study. Third, the instructions and measures of the questionnaire were translated into three languages, as well as available in English. Although care was taken to follow strict translation procedures, such as blinded back translation [37], it is possible that minor deviations between languages in the meaning of some words remained. Finally, our study was limited by only investigating DSM-5-defined PTSD symptoms. Despite evidence of the cross-cultural validity of DSM-defined PTSD, culturally specific responses to trauma also exist, such as somatic symptoms, which are not currently included in the DSM-5 criteria for PTSD [63]. Future ethnographic research is necessary to identify the full breadth of symptom experience for trauma-exposed refugees.

## Conclusions

Establishing an accurate conceptualisation of the latent structure of PTSD is essential to the development of effective assessment and treatment. The findings of the current study offer preliminary support for the applicability of the Anhedonia model to a culturally diverse refugee sample, and contribute to a growing body of studies which indicate that the DSM-5 model may not best represent the symptom structure of PTSD found across both western and non-western samples. In light of preliminary support for the six-factor Anhedonia model, an important area of future research is reconciling the trend of the DSM-5 literature, which favours more sophisticated models with more numerous factors, with research based on the tenth edition of the *International Statistical Classification of Diseases and Related Health Problems* (ICD-10) [64], which instead favours more simplified models with fewer symptom clusters. In fact, the draft proposal for the ICD-11 specifies two symptoms for each PTSD symptom cluster of re-experiencing, avoidance, and hyperarousal [50, 65]. As such, future research that balances the goals of phenomenological investigation with a need for parsimony and consideration of clinical resources may assist in translating research on the symptom structure of PTSD into a useful clinical tool appropriate to clinical settings. Notably, further studies are required to determine whether the findings of our study, which was conducted with a sample of traumatised refugees resettled in Australia, can be replicated within different refugee samples, such as refugees resettled in low or middle income countries as well as those who are internally displaced, currently in transition or residing in refugee camps.

## Abbreviations

DSM-5: the fifth edition of the Diagnostic and Statistical Manual and Mental Disorders
DSM-III: the third edition of the Diagnostic and Statistical Manual and Mental Disorders
DSM-IV: the fourth edition of the Diagnostic and Statistical Manual and Mental Disorders
NAMC: Negative alterations in mood and cognitions
PTE: Potentially traumatic event
PTSD: Post-traumatic stress disorder

## References

[1] Lindert J, Carta MG, Schäfer I, Mollica RF (2016). Refugees mental health-a public mental health challenge. Eur J Pub Health.

[2] Office of the United Nations High Commissioner for Refugees [UNHCR] (2016). Global trends report.

[3] Bogic M, Njoku A, Priebe S. Long-term mental health of war-refugees: a systematic literature review. BMC int health and human rights. 2015;15:29. 10.1186/s12914-015-0064-9 PMC4624599 26510473

[4] Fazel M, Wheeler J, Danesh J (2005). Prevalence of serious mental disorder in 7000 refugees resettled in western countries: a systematic review. Lancet.

[5] Steel Z, Chey T, Silove D, Marnane C, Bryant RA, Van Ommeren M (2009). Association of torture and other potentially traumatic events with mental health outcomes among populations exposed to mass conflict and displacement: a systematic review and meta-analysis. J Am Med Assoc.

[6] American Psychiatric Association. Diagnostic and statistical manual of mental disorders. Third ed. Washington, DC: American Psychiatric Association Press; 1980.

[7] Armour C, Mullerova J, Elhai JD (2016). A systematic literature review of PTSD's latent structure in the diagnostic and statistical manual of mental disorders: DSM-IV to DSM-5. Clin Psychol Rev.

[8] American Psychiatric Association. Diagnostic and statistical manual of mental disorders. Fifth ed. Washington, DC: American Psychiatric Association Press; 2013.

[9] Michalopoulos LM, Unick GK, Haroz EE, Bass J, Murray L, Bolton P (2015). Exploring the fit of western PTSD models across three non-western low- and middle income countries. Traumatology.

[10] Rasmussen A, Smith H, Keller AS (2007). Factor structure of PTSD symptoms among west and central African refugees. J Trauma Stress.

[11] Tay AK, Jayasuriya R, Jayasuriya D, Silove D (2017). Assessing the factorial structure and measurement invariance of PTSD by gender and ethnic groups in Sri Lanka: an analysis of the modified Harvard trauma questionnaire (HTQ). J Anxiety Disord.

[12] American Psychiatric Association. Diagnostic and statistical manual of mental disorders. Fourth ed. Washington, DC: American Psychiatric Association Press; 1994.

[13] Yufik T, Simms LJ (2010). A meta-analytic investigation of the structure of posttraumatic stress disorder symptoms. J Abnorm Psychol.

[14] King DW, Leskin GA, King LA, Weathers FW (1998). Confirmatory factor analysis of the clinician-administered PTSD scale: evidence for the dimensionality of posttraumatic stress disorder. Psychol Assess.

[15] Simms LJ, Watson D, Doebbelling BN (2002). Confirmatory factor analyses of posttraumatic stress symptoms in deployed and nondeployed veterans of the Gulf war. J Abnorm Psychol.

[16] Elhai JD, Biehn TL, Armour C, Klopper JJ, Frueh BC, Palmieri PA (2011). Evidence for a unique PTSD construct represented by PTSD's D1–D3 symptoms. J Anxiety Disord..

[17] Foa EB, Riggs DS, Gershuny BS (1995). Arousal, numbing, and intrusion: symptom structure of PTSD following assault. Am J Psychiatr.

[18] King LA, King DW (1994). Latent structure of the Mississippi scale for combat-related post-traumatic stress disorder: exploratory and higher-order confirmatory factor analyses. Assessment.

[19] Taylor S, Kuch K, Koch WJ, Crockett DJ, Passey G (1998). The structure of posttraumatic stress symptoms. J Abnorm Psychol.

[20] Pietrzak RH, Tsai J, Harpaz-Rotem I, Whealin JM, Southwick SM (2012). Support for a novel five-factor model of posttraumatic stress symptoms in three independent samples of Iraq/Afghanistan veterans: a confirmatory factor analytic study. J Psychiatr Res.

[21] Liu P, Wang L, Cao C (2014). The underlying dimensions of DSM-5 posttraumatic stress disorder symptoms in an epidemiological sample of Chinese earthquake survivors. J anxiety disord..

[22] Tsai J, Harpaz-Rotem I, Armour C, Southwick SM, Krystal JH, Pietrzak RH (2015). Dimensional structure of DSM-5 posttraumatic stress disorder symptoms: results from the National Health and resilience in veterans study. J clin psychiat.

[23] Cuthbert BN, Kozak MJ (2013). Constructing constructs for psychopathology: the NIMH research domain criteria. J Abnorm Psychol.

[24] Watson D (2009). Differentiating the mood and anxiety disorders: a quadripartite model. Annu Rev Clin Psychol.

[25] Watson D, Clark LA, Stasik SM (2011). Emotions and the emotional disorders: a quantitative hierarchical perspective. Int J Clin Health Psychol.

[26] Armour C, Tsai J, Durham TA (2015). Dimensional structure of DSM-5 posttraumatic stress symptoms: support for a hybrid anhedonia and externalizing behaviors model. J Psychiatr Res.

[27] Mordeno IG, Carpio JGE, Nalipay MJN, Saavedra RLJ (2017). PTSD's underlying dimensions in typhoon Haiyan survivors: assessing DSM-5 symptomatology-based PTSD models and their relation to posttraumatic cognition. Psychiatry Q.

[28] Osterman JE, De Jong JT (2007). Cultural issues and trauma. Handb PTSD Sci pract.

[29] Hollifield M, Warner TD, Lian N, Krakow B, Jenkins JH, Kesler J, Stevenson J, Westermeyer J (2002). Measuring trauma and health status in refugees: a critical review. JAMA.

[30] Nickerson A, Bryant RA, Silove D, Steel Z (2011). A critical review of psychological treatments of posttraumatic stress disorder in refugees. Clin Psychol Rev.

[31] Li SSY, Liddell BJ, Nickerson A (2016). The relationship between post-migration stress and psychological disorders in refugees and asylum seekers. Curr Psychiatry Rep.

[32] Palmieri PA, Marshall GN, Schell TL (2007). Confirmatory factor analysis of posttraumatic stress symptoms in Cambodian refugees. J Trauma Stress.

[33] Tay AK, Rees S, Chen J, Kareth M, Silove D (2015). The structure of post-traumatic stress disorder and complex post-traumatic stress disorder amongst west Papuan refugees. BMC psychiatry..

[34] Vindbjerg E, Carlsson J, Mortensen EL, Elklit A, Makransky G (2016). The latent structure of post-traumatic stress disorder among Arabic-speaking refugees receiving psychiatric treatment in Denmark. BMC psychiatry.

[35] Vinson GA, Chang Z (2012). PTSD symptom structure among west African war trauma survivors living in African refugee camps: a factor-analytic investigation. J Trauma Stress.

[36] Schnyder U, Müller J, Morina N, Schick M, Bryant RA, Nickerson A (2015). A comparison of DSM-5 and DSM-IV diagnostic criteria for posttraumatic stress disorder in traumatized refugees. J Trauma Stress.

[37] World Health Organization. Process of translation and adaptation of instruments. http://www.who.int/substance_abuse/research_tools/translation/en/ Accessed 6 Sept 2017.

[38] Mollica RF, Caspi-Yavin Y, Bollini P, Truong T, Tor S, Lavelle J (1992). The Harvard trauma questionnaire: validating a cross-cultural instrument for measuring torture, trauma, and posttraumatic stress disorder in Indochinese refugees. J Nerv Ment Dis.

[39] Foa EB, Cashman L, Jaycox L, Perry K (1997). The validation of a self-report measure of posttraumatic stress disorder: the posttraumatic diagnostic scale. Psychol Assess.

[40] Preacher KJ, Coffman DL. Computing power and minimum sample size for RMSEA [computer software]. Available from http://quantpsy.org/ (2006, May) Accessed 18 Jan 2018.

[41] Muthén LK, Muthén BO (2017). Mplus user's guide.

[42] Flora DB, Curran PJ (2004). An empirical evaluation of alternative methods of estimation for confirmatory factor analysis with ordinal data. Psychol Methods.

[43] Wirth RJ, Edwards MC (2007). Item factor analysis: current approaches and future directions. Psychol Methods.

[44] Brown TA (2006). Confirmatory factor analysis for applied research.

[45] Steiger JH (1989). EzPATH: causal modelling.

[46] Bentler PM (1990). Comparative fit indexes in structural models. Psychol Bull.

[47] Tucker LR, Lewis C (1973). A reliability coefficient for maximum likelihood factor analysis. Psychometrika.

[48] Kline RB (2010). Principles and practice of structural equation modeling.

[49] Yu CY (2002). Evaluating cutoff criteria of model fit indices for latent variable models with binary and continuous outcomes.

[50] World Health Organization: ICD-11 Beta draft. 2015. https://icd.who.int/dev11/l-m/en. Accessed 25 Aug 2017.

[51] Marsh HW, Hau K, Balla JR, Grayson D (1998). Is more ever too much? The number of indicators per factor in confirmatory factor analysis. Multivar Behav Res.

[52] Armour C, Carragher N, Elhai JD (2013). Assessing the fit of the dysphoric arousal model across two nationally representative epidemiological surveys: the Australian NSMHWB and the United States NESARC. J anxiety disord.

[53] Armour C, Raudzah Ghazali S, Elklit A (2013). PTSD's latent structure in Malaysian tsunami victims: assessing the newly proposed dysphoric arousal model. Psychiatry Res.

[54] Pietrzak RH, Feder A, Schechter CB (2014). Dimensional structure and course of post-traumatic stress symptomatology in world trade center responders. Psychol Med.

[55] Wang R, Wang L, Li Z, Cao C, Shi Z, Zhang J (2013). Latent structure of posttraumatic stress disorder symptoms in an adolescent sample one month after an earthquake. J Adolesc.

[56] Watson D (2005). Rethinking the mood and anxiety disorders: a quantitative hierarchical model for DSM-V. J Abnorm Psychol.

[57] Forbes D, Lockwood E, Elhai JD (2015). An evaluation of the DSM-5 factor structure for posttraumatic stress disorder in survivors of traumatic injury. J anxiety disord..

[58] Forbes D, Fletcher S, Parslow R (2012). Trauma at the hands of another: longitudinal study of differences in the posttraumatic stress disorder symptom profile following interpersonal compared with noninterpersonal trauma. J clin psychiatry.

[59] Miller MW, Wolf EJ, Kilpatrick D (2013). The prevalence and latent structure of proposed DSM-5 posttraumatic stress disorder symptoms in U.S. national and veteran samples. Psychol TraumaTheo Res, Prac Pol.

[60] Cohen J (2008). Safe in our hands?: a study of suicide and self-harm in asylum seekers. J Forensic Legal Med.

[61] Patel N, Hodes M (2006). Violent deliberate self-harm amongst adolescent refugees. Euro Child Adolescent Psychiatry.

[62] Blanchard EB, Jones-Alexander J, Buckley TC, Forneris CA (1996). Psychometric properties of the PTSD checklist (PCL). Behav Res Ther.

[63] Hinton DE, Lewis-Fernandez R (2011). The cross-cultural validity of posttraumatic stress disorder: implications for DSM-5. Depress Anxiety.

[64] World Health Organization (1992). The ICD-10 classification of mental and Behavioural disorders: clinical descriptions and diagnostic guidelines.

[65] Maercker A, Brewin CR, Bryant RA (2013). Diagnosis and classification of disorders specifically associated with stress: proposals for ICD-11. World Psychiatry.

